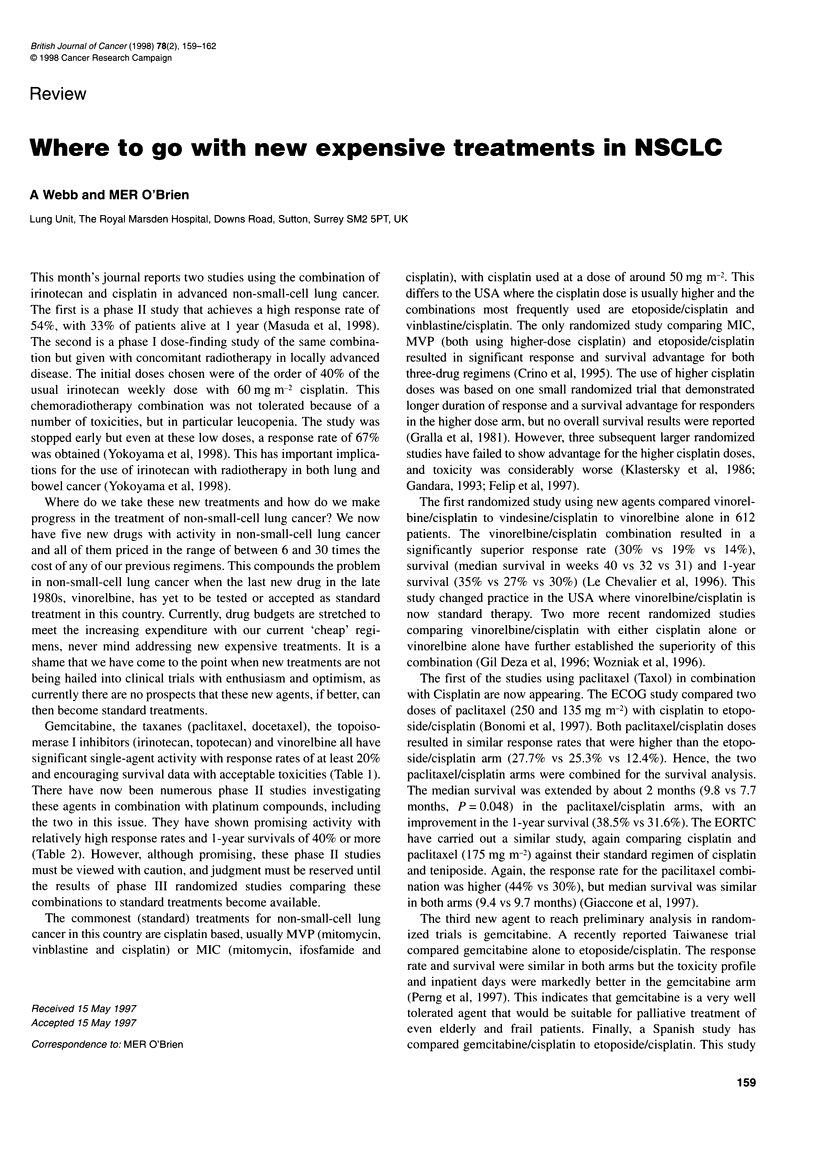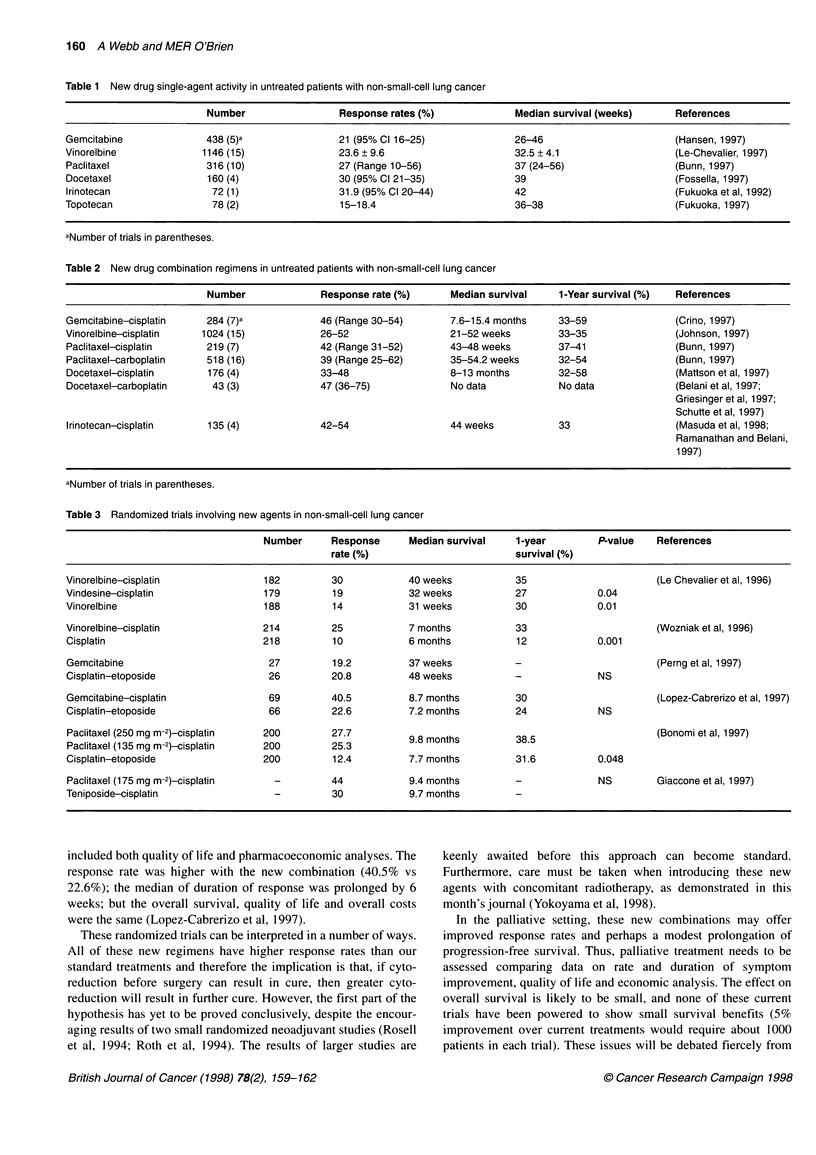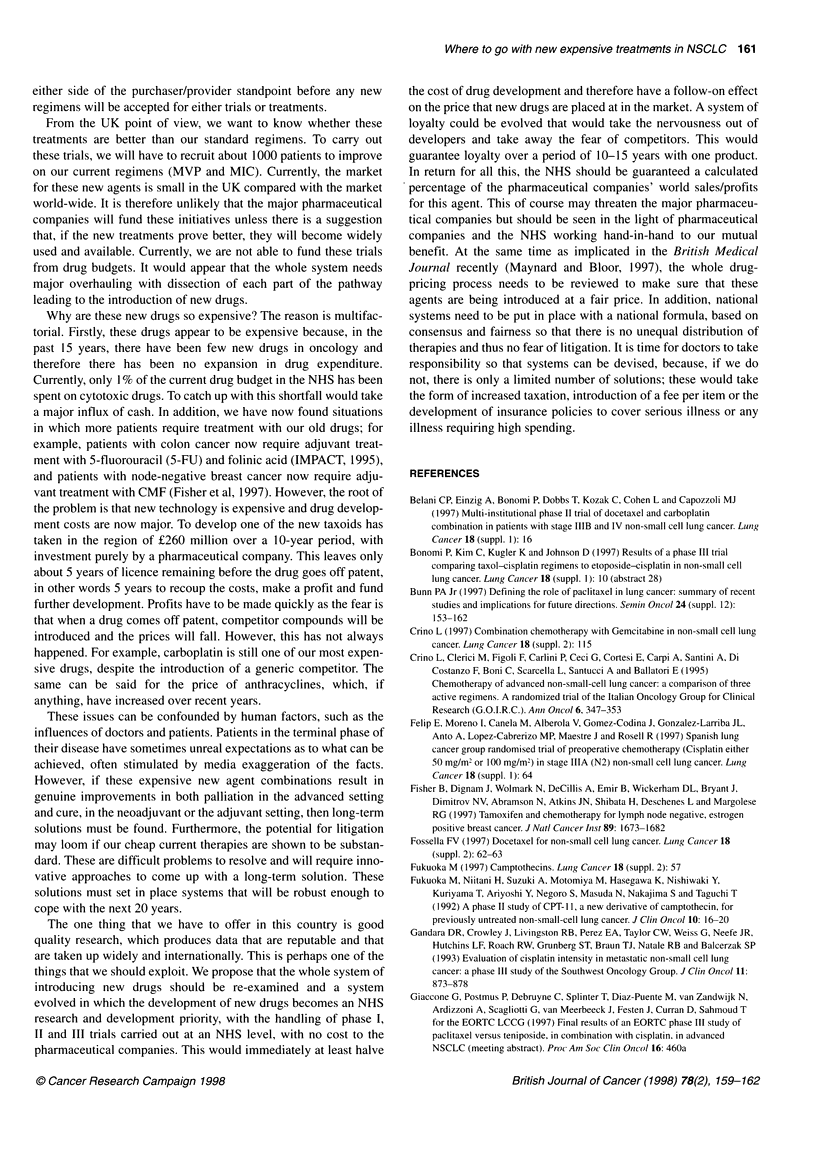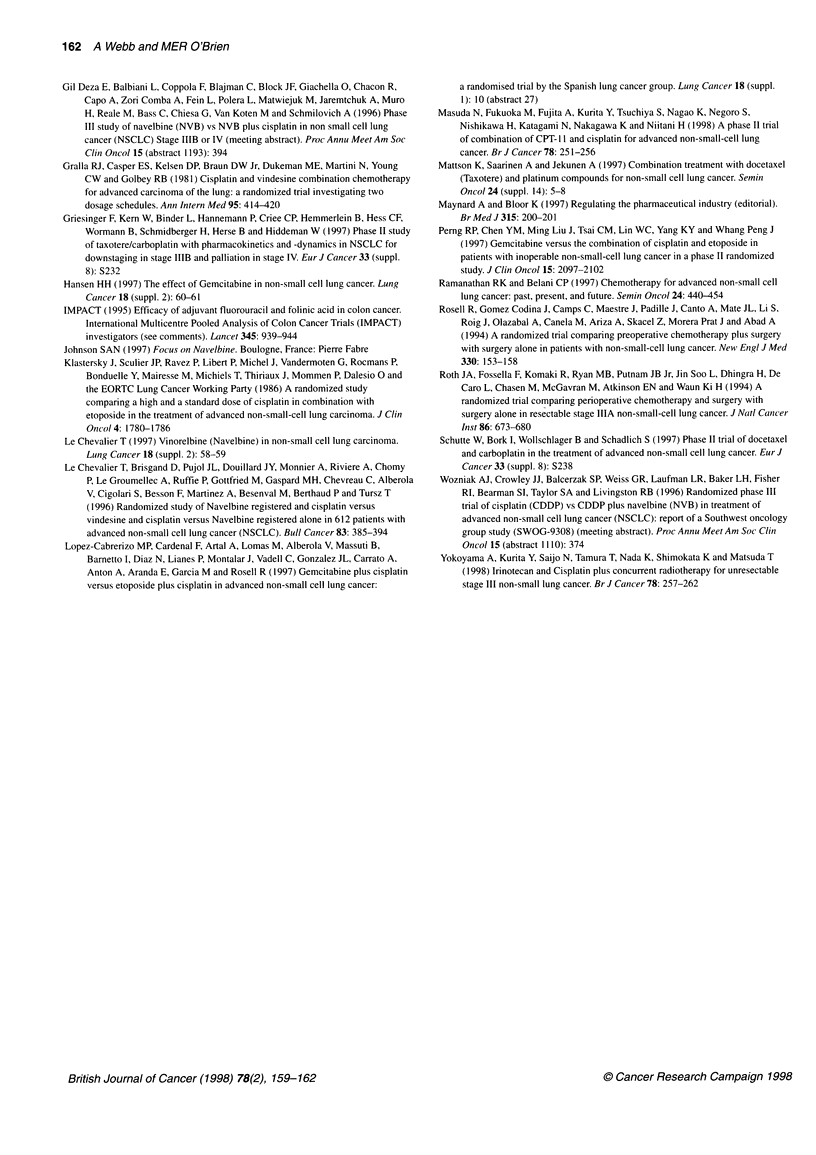# Where to go with new expensive treatments in NSCLC.

**DOI:** 10.1038/bjc.1998.458

**Published:** 1998-07

**Authors:** A. Webb, M. E. O'Brien

**Affiliations:** Lung Unit, The Royal Marsden Hospital, Sutton, Surrey, UK.


British Journal of Cancer (1998) 78(2), 159–162
© 1998 Cancer Research Campaign

Review

### Where to go with new expensive treatments in NSCLC

**A Webb and MER O'Brien**

Lung Unit, The Royal Marsden Hospital, Downs Road, Sutton, Surrey SM2 5PT, UK

This month's journal reports two studies using the combination of irinotecan and cisplatin in advanced non-small-cell lung cancer. The first is a phase II study that achieves a high response rate of 54%, with 33% of patients alive at 1 year (Masuda et al, 1998). The second is a phase I dose-finding study of the same combination but given with concomitant radiotherapy in locally advanced disease. The initial doses chosen were of the order of 40% of the usual irinotecan weekly dose with 60 mg $m^{-2}$ cisplatin. This chemoradiotherapy combination was not tolerated because of a number of toxicities, but in particular leucopenia. The study was stopped early but even at these low doses, a response rate of 67% was obtained (Yokoyama et al, 1998). This has important implications for the use of irinotecan with radiotherapy in both lung and bowel cancer (Yokoyama et al, 1998).

Where do we take these new treatments and how do we make progress in the treatment of non-small-cell lung cancer? We now have five new drugs with activity in non-small-cell lung cancer and all of them priced in the range of between 6 and 30 times the cost of any of our previous regimens. This compounds the problem in non-small-cell lung cancer when the last new drug in the late 1980s, vinorelbine, has yet to be tested or accepted as standard treatment in this country. Currently, drug budgets are stretched to meet the increasing expenditure with our current 'cheap' regimens, never mind addressing new expensive treatments. It is a shame that we have come to the point when new treatments are not being hailed into clinical trials with enthusiasm and optimism, as currently there are no prospects that these new agents, if better, can then become standard treatments.

Gemcitabine, the taxanes (paclitaxel, docetaxel), the topoisomerase I inhibitors (irinotecan, topotecan) and vinorelbine all have significant single-agent activity with response rates of at least 20% and encouraging survival data with acceptable toxicities (Table 1). There have now been numerous phase II studies investigating these agents in combination with platinum compounds, including the two in this issue. They have shown promising activity with relatively high response rates and 1-year survivals of 40% or more (Table 2). However, although promising, these phase II studies must be viewed with caution, and judgment must be reserved until the results of phase III randomized studies comparing these combinations to standard treatments become available.

The commonest (standard) treatments for non-small-cell lung cancer in this country are cisplatin based, usually MVP (mitomycin, vinblastine and cisplatin) or MIC (mitomycin, ifosfamide and cisplatin), with cisplatin used at a dose of around 50 mg $m^{-2}$. This differs to the USA where the cisplatin dose is usually higher and the combinations most frequently used are etoposide/cisplatin and vinblastine/cisplatin. The only randomized study comparing MIC, MVP (both using higher-dose cisplatin) and etoposide/cisplatin resulted in significant response and survival advantage for both three-drug regimens (Crino et al, 1995). The use of higher cisplatin doses was based on one small randomized trial that demonstrated longer duration of response and a survival advantage for responders in the higher dose arm, but no overall survival results were reported (Gralla et al, 1981). However, three subsequent larger randomized studies have failed to show advantage for the higher cisplatin doses, and toxicity was considerably worse (Klastersky et al, 1986; Gandara, 1993; Felip et al, 1997).

The first randomized study using new agents compared vinorelbine/cisplatin to vindesine/cisplatin to vinorelbine alone in 612 patients. The vinorelbine/cisplatin combination resulted in a significantly superior response rate (30% vs 19% vs 14%), survival (median survival in weeks 40 vs 32 vs 31) and 1-year survival (35% vs 27% vs 30%) (Le Chevalier et al, 1996). This study changed practice in the USA where vinorelbine/cisplatin is now standard therapy. Two more recent randomized studies comparing vinorelbine/cisplatin with either cisplatin alone or vinorelbine alone have further established the superiority of this combination (Gil Deza et al, 1996; Wozniak et al, 1996).

The first of the studies using paclitaxel (Taxol) in combination with Cisplatin are now appearing. The ECOG study compared two doses of paclitaxel (250 and 135 mg $m^{-2}$) with cisplatin to etoposide/cisplatin (Bonomi et al, 1997). Both paclitaxel/cisplatin doses resulted in similar response rates that were higher than the etoposide/cisplatin arm (27.7% vs 25.3% vs 12.4%). Hence, the two paclitaxel/cisplatin arms were combined for the survival analysis. The median survival was extended by about 2 months (9.8 vs 7.7 months, $P = 0.048$) in the paclitaxel/cisplatin arms, with an improvement in the 1-year survival (38.5% vs 31.6%). The EORTC have carried out a similar study, again comparing cisplatin and paclitaxel (175 mg $m^{-2}$) against their standard regimen of cisplatin and teniposide. Again, the response rate for the pacilitaxel combination was higher (44% vs 30%), but median survival was similar in both arms (9.4 vs 9.7 months) (Giaccone et al, 1997).

The third new agent to reach preliminary analysis in randomized trials is gemcitabine. A recently reported Taiwanese trial compared gemcitabine alone to etoposide/cisplatin. The response rate and survival were similar in both arms but the toxicity profile and inpatient days were markedly better in the gemcitabine arm (Perng et al, 1997). This indicates that gemcitabine is a very well tolerated agent that would be suitable for palliative treatment of even elderly and frail patients. Finally, a Spanish study has compared gemcitabine/cisplatin to etoposide/cisplatin. This study

*Received 15 May 1997*
*Accepted 15 May 1997*

*Correspondence to:* MER O'Brien

**Table 1** New drug single-agent activity in untreated patients with non-small-cell lung cancer

| | Number | Response rates (%) | Median survival (weeks) | References |
|---|---|---|---|---|
| Gemcitabine | 438 (5)[a] | 21 (95% CI 16–25) | 26–46 | (Hansen, 1997) |
| Vinorelbine | 1146 (15) | 23.6 ± 9.6 | 32.5 ± 4.1 | (Le-Chevalier, 1997) |
| Paclitaxel | 316 (10) | 27 (Range 10–56) | 37 (24–56) | (Bunn, 1997) |
| Docetaxel | 160 (4) | 30 (95% CI 21–35) | 39 | (Fossella, 1997) |
| Irinotecan | 72 (1) | 31.9 (95% CI 20–44) | 42 | (Fukuoka et al, 1992) |
| Topotecan | 78 (2) | 15–18.4 | 36–38 | (Fukuoka, 1997) |

[a]Number of trials in parentheses.

**Table 2** New drug combination regimens in untreated patients with non-small-cell lung cancer

| | Number | Response rate (%) | Median survival | 1-Year survival (%) | References |
|---|---|---|---|---|---|
| Gemcitabine–cisplatin | 284 (7)[a] | 46 (Range 30–54) | 7.6–15.4 months | 33–59 | (Crino, 1997) |
| Vinorelbine–cisplatin | 1024 (15) | 26–52 | 21–52 weeks | 33–35 | (Johnson, 1997) |
| Paclitaxel–cisplatin | 219 (7) | 42 (Range 31–52) | 43–48 weeks | 37–41 | (Bunn, 1997) |
| Paclitaxel–carboplatin | 518 (16) | 39 (Range 25–62) | 35–54.2 weeks | 32–54 | (Bunn, 1997) |
| Docetaxel–cisplatin | 176 (4) | 33–48 | 8–13 months | 32–58 | (Mattson et al, 1997) |
| Docetaxel–carboplatin | 43 (3) | 47 (36–75) | No data | No data | (Belani et al, 1997; Griesinger et al, 1997; Schutte et al, 1997) |
| Irinotecan–cisplatin | 135 (4) | 42–54 | 44 weeks | 33 | (Masuda et al, 1998; Ramanathan and Belani, 1997) |

[a]Number of trials in parentheses.

**Table 3** Randomized trials involving new agents in non-small-cell lung cancer

| | Number | Response rate (%) | Median survival | 1-year survival (%) | *P*-value | References |
|---|---|---|---|---|---|---|
| Vinorelbine–cisplatin | 182 | 30 | 40 weeks | 35 | | (Le Chevalier et al, 1996) |
| Vindesine–cisplatin | 179 | 19 | 32 weeks | 27 | 0.04 | |
| Vinorelbine | 188 | 14 | 31 weeks | 30 | 0.01 | |
| Vinorelbine–cisplatin | 214 | 25 | 7 months | 33 | | (Wozniak et al, 1996) |
| Cisplatin | 218 | 10 | 6 months | 12 | 0.001 | |
| Gemcitabine | 27 | 19.2 | 37 weeks | – | | (Perng et al, 1997) |
| Cisplatin–etoposide | 26 | 20.8 | 48 weeks | – | NS | |
| Gemcitabine–cisplatin | 69 | 40.5 | 8.7 months | 30 | | (Lopez-Cabrerizo et al, 1997) |
| Cisplatin–etoposide | 66 | 22.6 | 7.2 months | 24 | NS | |
| Paclitaxel (250 mg $m^{-2}$)–cisplatin | 200 | 27.7 | 9.8 months | 38.5 | | (Bonomi et al, 1997) |
| Paclitaxel (135 mg $m^{-2}$)–cisplatin | 200 | 25.3 | | | | |
| Cisplatin–etoposide | 200 | 12.4 | 7.7 months | 31.6 | 0.048 | |
| Paclitaxel (175 mg $m^{-2}$)–cisplatin | – | 44 | 9.4 months | – | NS | Giaccone et al, 1997) |
| Teniposide–cisplatin | – | 30 | 9.7 months | – | | |

included both quality of life and pharmacoeconomic analyses. The response rate was higher with the new combination (40.5% vs 22.6%); the median of duration of response was prolonged by 6 weeks; but the overall survival, quality of life and overall costs were the same (Lopez-Cabrerizo et al, 1997).

These randomized trials can be interpreted in a number of ways. All of these new regimens have higher response rates than our standard treatments and therefore the implication is that, if cyto-reduction before surgery can result in cure, then greater cyto-reduction will result in further cure. However, the first part of the hypothesis has yet to be proved conclusively, despite the encouraging results of two small randomized neoadjuvant studies (Rosell et al, 1994; Roth et al, 1994). The results of larger studies are keenly awaited before this approach can become standard. Furthermore, care must be taken when introducing these new agents with concomitant radiotherapy, as demonstrated in this month's journal (Yokoyama et al, 1998).

In the palliative setting, these new combinations may offer improved response rates and perhaps a modest prolongation of progression-free survival. Thus, palliative treatment needs to be assessed comparing data on rate and duration of symptom improvement, quality of life and economic analysis. The effect on overall survival is likely to be small, and none of these current trials have been powered to show small survival benefits (5% improvement over current treatments would require about 1000 patients in each trial). These issues will be debated fiercely from

 © Cancer Research Campaign 1998

either side of the purchaser/provider standpoint before any new regimens will be accepted for either trials or treatments.

From the UK point of view, we want to know whether these treatments are better than our standard regimens. To carry out these trials, we will have to recruit about 1000 patients to improve on our current regimens (MVP and MIC). Currently, the market for these new agents is small in the UK compared with the market world-wide. It is therefore unlikely that the major pharmaceutical companies will fund these initiatives unless there is a suggestion that, if the new treatments prove better, they will become widely used and available. Currently, we are not able to fund these trials from drug budgets. It would appear that the whole system needs major overhauling with dissection of each part of the pathway leading to the introduction of new drugs.

Why are these new drugs so expensive? The reason is multifactorial. Firstly, these drugs appear to be expensive because, in the past 15 years, there have been few new drugs in oncology and therefore there has been no expansion in drug expenditure. Currently, only 1% of the current drug budget in the NHS has been spent on cytotoxic drugs. To catch up with this shortfall would take a major influx of cash. In addition, we have now found situations in which more patients require treatment with our old drugs; for example, patients with colon cancer now require adjuvant treatment with 5-fluorouracil (5-FU) and folinic acid (IMPACT, 1995), and patients with node-negative breast cancer now require adjuvant treatment with CMF (Fisher et al, 1997). However, the root of the problem is that new technology is expensive and drug development costs are now major. To develop one of the new taxoids has taken in the region of £260 million over a 10-year period, with investment purely by a pharmaceutical company. This leaves only about 5 years of licence remaining before the drug goes off patent, in other words 5 years to recoup the costs, make a profit and fund further development. Profits have to be made quickly as the fear is that when a drug comes off patent, competitor compounds will be introduced and the prices will fall. However, this has not always happened. For example, carboplatin is still one of our most expensive drugs, despite the introduction of a generic competitor. The same can be said for the price of anthracyclines, which, if anything, have increased over recent years.

These issues can be confounded by human factors, such as the influences of doctors and patients. Patients in the terminal phase of their disease have sometimes unreal expectations as to what can be achieved, often stimulated by media exaggeration of the facts. However, if these expensive new agent combinations result in genuine improvements in both palliation in the advanced setting and cure, in the neoadjuvant or the adjuvant setting, then long-term solutions must be found. Furthermore, the potential for litigation may loom if our cheap current therapies are shown to be substandard. These are difficult problems to resolve and will require innovative approaches to come up with a long-term solution. These solutions must set in place systems that will be robust enough to cope with the next 20 years.

The one thing that we have to offer in this country is good quality research, which produces data that are reputable and that are taken up widely and internationally. This is perhaps one of the things that we should exploit. We propose that the whole system of introducing new drugs should be re-examined and a system evolved in which the development of new drugs becomes an NHS research and development priority, with the handling of phase I, II and III trials carried out at an NHS level, with no cost to the pharmaceutical companies. This would immediately at least halve the cost of drug development and therefore have a follow-on effect on the price that new drugs are placed at in the market. A system of loyalty could be evolved that would take the nervousness out of developers and take away the fear of competitors. This would guarantee loyalty over a period of 10–15 years with one product. In return for all this, the NHS should be guaranteed a calculated percentage of the pharmaceutical companies' world sales/profits for this agent. This of course may threaten the major pharmaceutical companies but should be seen in the light of pharmaceutical companies and the NHS working hand-in-hand to our mutual benefit. At the same time as implicated in the *British Medical Journal* recently (Maynard and Bloor, 1997), the whole drug-pricing process needs to be reviewed to make sure that these agents are being introduced at a fair price. In addition, national systems need to be put in place with a national formula, based on consensus and fairness so that there is no unequal distribution of therapies and thus no fear of litigation. It is time for doctors to take responsibility so that systems can be devised, because, if we do not, there is only a limited number of solutions; these would take the form of increased taxation, introduction of a fee per item or the development of insurance policies to cover serious illness or any illness requiring high spending.

### REFERENCES

Belani CP, Einzig A, Bonomi P, Dobbs T, Kozak C, Cohen L and Capozzoli MJ (1997) Multi-institutional phase II trial of docetaxel and carboplatin combination in patients with stage IIIB and IV non-small cell lung cancer. *Lung Cancer* **18** (suppl. 1): 16

Bonomi P, Kim C, Kugler K and Johnson D (1997) Results of a phase III trial comparing taxol–cisplatin regimens to etoposide–cisplatin in non-small cell lung cancer. *Lung Cancer* **18** (suppl. 1): 10 (abstract 28)

Bunn PA Jr (1997) Defining the role of paclitaxel in lung cancer: summary of recent studies and implications for future directions. *Semin Oncol* **24** (suppl. 12): 153–162

Crino L (1997) Combination chemotherapy with Gemcitabine in non-small cell lung cancer. *Lung Cancer* **18** (suppl. 2): 115

Crino L, Clerici M, Figoli F, Carlini P, Ceci G, Cortesi E, Carpi A, Santini A, Di Costanzo F, Boni C, Scarcella L, Santucci A and Ballatori E (1995) Chemotherapy of advanced non-small-cell lung cancer: a comparison of three active regimens. A randomized trial of the Italian Oncology Group for Clinical Research (G.O.I.R.C.). *Ann Oncol* **6**, 347–353

Felip E, Moreno I, Canela M, Alberola V, Gomez-Codina J, Gonzalez-Larriba JL, Anto A, Lopez-Cabrerizo MP, Maestre J and Rosell R (1997) Spanish lung cancer group randomised trial of preoperative chemotherapy (Cisplatin either 50 mg/m$^2$ or 100 mg/m$^2$) in stage IIIA (N2) non-small cell lung cancer. *Lung Cancer* **18** (suppl. 1): 64

Fisher B, Dignam J, Wolmark N, DeCillis A, Emir B, Wickerham DL, Bryant J, Dimitrov NV, Abramson N, Atkins JN, Shibata H, Deschenes L and Margolese RG (1997) Tamoxifen and chemotherapy for lymph node negative, estrogen positive breast cancer. *J Natl Cancer Inst* **89**: 1673–1682

Fossella FV (1997) Docetaxel for non-small cell lung cancer. *Lung Cancer* **18** (suppl. 2): 62–63

Fukuoka M (1997) Camptothecins. *Lung Cancer* **18** (suppl. 2): 57

Fukuoka M, Niitani H, Suzuki A, Motomiya M, Hasegawa K, Nishiwaki Y, Kuriyama T, Ariyoshi Y, Negoro S, Masuda N, Nakajima S and Taguchi T (1992) A phase II study of CPT-11, a new derivative of camptothecin, for previously untreated non-small-cell lung cancer. *J Clin Oncol* **10**: 16–20

Gandara DR, Crowley J, Livingston RB, Perez EA, Taylor CW, Weiss G, Neefe JR, Hutchins LF, Roach RW, Grunberg ST, Braun TJ, Natale RB and Balcerzak SP (1993) Evaluation of cisplatin intensity in metastatic non-small cell lung cancer: a phase III study of the Southwest Oncology Group. *J Clin Oncol* **11**: 873–878

Giaccone G, Postmus P, Debruyne C, Splinter T, Diaz-Puente M, van Zandwijk N, Ardizzoni A, Scagliotti G, van Meerbeeck J, Festen J, Curran D, Sahmoud T for the EORTC LCCG (1997) Final results of an EORTC phase III study of paclitaxel versus teniposide, in combination with cisplatin, in advanced NSCLC (meeting abstract). *Proc Am Soc Clin Oncol* **16**: 460a

© Cancer Research Campaign 1998 

Gil Deza E, Balbiani L, Coppola F, Blajman C, Block JF, Giachella O, Chacon R, Capo A, Zori Comba A, Fein L, Polera L, Matwiejuk M, Jaremtchuk A, Muro H, Reale M, Bass C, Chiesa G, Van Koten M and Schmilovich A (1996) Phase III study of navelbine (NVB) vs NVB plus cisplatin in non small cell lung cancer (NSCLC) Stage IIIB or IV (meeting abstract). *Proc Annu Meet Am Soc Clin Oncol* **15** (abstract 1193): 394

Gralla RJ, Casper ES, Kelsen DP, Braun DW Jr, Dukeman ME, Martini N, Young CW and Golbey RB (1981) Cisplatin and vindesine combination chemotherapy for advanced carcinoma of the lung: a randomized trial investigating two dosage schedules. *Ann Intern Med* **95**: 414–420

Griesinger F, Kern W, Binder L, Hannemann P, Criee CP, Hemmerlein B, Hess CF, Wormann B, Schmidberger H, Herse B and Hiddeman W (1997) Phase II study of taxotere/carboplatin with pharmacokinetics and -dynamics in NSCLC for downstaging in stage IIIB and palliation in stage IV. *Eur J Cancer* **33** (suppl. 8): S232

Hansen HH (1997) The effect of Gemcitabine in non-small cell lung cancer. *Lung Cancer* **18** (suppl. 2): 60–61

IMPACT (1995) Efficacy of adjuvant fluorouracil and folinic acid in colon cancer. International Multicentre Pooled Analysis of Colon Cancer Trials (IMPACT) investigators (see comments). *Lancet* **345**: 939–944

Johnson SAN (1997) *Focus on Navelbine*. Boulogne, France: Pierre Fabre

Klastersky J, Sculier JP, Ravez P, Libert P, Michel J, Vandermoten G, Rocmans P, Bonduelle Y, Mairesse M, Michiels T, Thiriaux J, Mommen P, Dalesio O and the EORTC Lung Cancer Working Party (1986) A randomized study comparing a high and a standard dose of cisplatin in combination with etoposide in the treatment of advanced non-small-cell lung carcinoma. *J Clin Oncol* **4**: 1780–1786

Le Chevalier T (1997) Vinorelbine (Navelbine) in non-small cell lung carcinoma. *Lung Cancer* **18** (suppl. 2): 58–59

Le Chevalier T, Brisgand D, Pujol JL, Douillard JY, Monnier A, Riviere A, Chomy P, Le Groumellec A, Ruffie P, Gottfried M, Gaspard MH, Chevreau C, Alberola V, Cigolari S, Besson F, Martinez A, Besenval M, Berthaud P and Tursz T (1996) Randomized study of Navelbine registered and cisplatin versus vindesine and cisplatin versus Navelbine registered alone in 612 patients with advanced non-small cell lung cancer (NSCLC). *Bull Cancer* **83**: 385–394

Lopez-Cabrerizo MP, Cardenal F, Artal A, Lomas M, Alberola V, Massuti B, Barnetto I, Diaz N, Lianes P, Montalar J, Vadell C, Gonzalez JL, Carrato A, Anton A, Aranda E, Garcia M and Rosell R (1997) Gemcitabine plus cisplatin versus etoposide plus cisplatin in advanced non-small cell lung cancer: a randomised trial by the Spanish lung cancer group. *Lung Cancer* **18** (suppl. 1): 10 (abstract 27)

Masuda N, Fukuoka M, Fujita A, Kurita Y, Tsuchiya S, Nagao K, Negoro S, Nishikawa H, Katagami N, Nakagawa K and Niitani H (1998) A phase II trial of combination of CPT-11 and cisplatin for advanced non-small-cell lung cancer. *Br J Cancer* **78**: 251–256

Mattson K, Saarinen A and Jekunen A (1997) Combination treatment with docetaxel (Taxotere) and platinum compounds for non-small cell lung cancer. *Semin Oncol* **24** (suppl. 14): 5–8

Maynard A and Bloor K (1997) Regulating the pharmaceutical industry (editorial). *Br Med J* **315**: 200–201

Perng RP, Chen YM, Ming Liu J, Tsai CM, Lin WC, Yang KY and Whang Peng J (1997) Gemcitabine versus the combination of cisplatin and etoposide in patients with inoperable non-small-cell lung cancer in a phase II randomized study. *J Clin Oncol* **15**: 2097–2102

Ramanathan RK and Belani CP (1997) Chemotherapy for advanced non-small cell lung cancer: past, present, and future. *Semin Oncol* **24**: 440–454

Rosell R, Gomez Codina J, Camps C, Maestre J, Padille J, Canto A, Mate JL, Li S, Roig J, Olazabal A, Canela M, Ariza A, Skacel Z, Morera Prat J and Abad A (1994) A randomized trial comparing preoperative chemotherapy plus surgery with surgery alone in patients with non-small-cell lung cancer. *New Engl J Med* **330**: 153–158

Roth JA, Fossella F, Komaki R, Ryan MB, Putnam JB Jr, Jin Soo L, Dhingra H, De Caro L, Chasen M, McGavran M, Atkinson EN and Waun Ki H (1994) A randomized trial comparing perioperative chemotherapy and surgery with surgery alone in resectable stage IIIA non-small-cell lung cancer. *J Natl Cancer Inst* **86**: 673–680

Schutte W, Bork I, Wollschlager B and Schadlich S (1997) Phase II trial of docetaxel and carboplatin in the treatment of advanced non-small cell lung cancer. *Eur J Cancer* **33** (suppl. 8): S238

Wozniak AJ, Crowley JJ, Balcerzak SP, Weiss GR, Laufman LR, Baker LH, Fisher RI, Bearman SI, Taylor SA and Livingston RB (1996) Randomized phase III trial of cisplatin (CDDP) vs CDDP plus navelbine (NVB) in treatment of advanced non-small cell lung cancer (NSCLC): report of a Southwest oncology group study (SWOG-9308) (meeting abstract). *Proc Annu Meet Am Soc Clin Oncol* **15** (abstract 1110): 374

Yokoyama A, Kurita Y, Saijo N, Tamura T, Nada K, Shimokata K and Matsuda T (1998) Irinotecan and Cisplatin plus concurrent radiotherapy for unresectable stage III non-small lung cancer. *Br J Cancer* **78**: 257–262

© Cancer Research Campaign 1998